# Semi-automatic analysis of standard uptake values in serial PET/CT studies in patients with lung cancer and lymphoma

**DOI:** 10.1186/1471-2342-12-6

**Published:** 2012-04-02

**Authors:** John Ly, Sabine Garpered, Peter Höglund, Eskil Jönsson, Sven Valind, Lars Edenbrandt, Per Wollmer

**Affiliations:** 1 Department of Clinical Sciences, Skåne University, Malmö, Sweden; 2 Competence Centre for Clin Research, Lund University, Lund, Sweden; 3 Department of Molecular and Clinical Medicine, Sahlgrenska Academy, Gothenburg University, Gothenburg, Sweden; 4 Clinical Physiology and Nuclear Medicine, Diagnostic Centre, Skåne University Hospital Malmö, SE-20502 Malmö, Sweden

**Keywords:** Image analysis, Radionuclide imaging, Quantification

## Abstract

**Background:**

Changes in maximum standardised uptake values (SUV_max_) between serial PET/CT studies are used to determine disease progression or regression in oncologic patients. To measure these changes manually can be time consuming in a clinical routine. A semi-automatic method for calculation of SUV_max _in serial PET/CT studies was developed and compared to a conventional manual method. The semi-automatic method first aligns the serial PET/CT studies based on the CT images. Thereafter, the reader selects an abnormal lesion in one of the PET studies. After this manual step, the program automatically detects the corresponding lesion in the other PET study, segments the two lesions and calculates the SUV_max _in both studies as well as the difference between the SUV_max _values. The results of the semi-automatic analysis were compared to that of a manual SUV_max _analysis using a Philips PET/CT workstation. Three readers did the SUV_max _readings in both methods. Sixteen patients with lung cancer or lymphoma who had undergone two PET/CT studies were included. There were a total of 26 lesions.

**Results:**

Linear regression analysis of changes in SUV_max _show that intercepts and slopes are close to the line of identity for all readers (reader 1: intercept = 1.02, R^2 ^= 0.96; reader 2: intercept = 0.97, R^2 ^= 0.98; reader 3: intercept = 0.99, R^2 ^= 0.98). Manual and semi-automatic method agreed in all cases whether SUV_max _had increased or decreased between the serial studies. The average time to measure SUV_max _changes in two serial PET/CT examinations was four to five times longer for the manual method compared to the semi-automatic method for all readers (reader 1: 53.7 vs. 10.5 s; reader 2: 27.3 vs. 6.9 s; reader 3: 47.5 vs. 9.5 s; *p *< 0.001 for all).

**Conclusions:**

Good agreement was shown in assessment of SUV_max _changes between manual and semi-automatic method. The semi-automatic analysis was four to five times faster to perform than the manual analysis. These findings show the feasibility of using semi-automatic methods for calculation of SUV_max _in clinical routine and encourage further development of programs using this type of methods.

## Background

Changes in maximum standard uptake values (SUV_max_) and computed tomography (CT) tumour size in ^18^FDG positron emission tomography (PET)/CT follow-up studies in oncologic patients are often assessed in tandem to determine disease progression or regression. RECIST criteria based on CT [1] are most frequently used, but criteria based on the uptake of ^18^FDG are receiving increasing attention [2,3]. With this, the demand for quantitative monitoring of SUV has grown as the indications for performing follow-up ^18^FDG PET/CT are expanding [4]. SUV_max _measurements are more readily reproducible than CT sizes in both pre- and post-treatment studies [5]. Currently available PET/CT software enables the use of tools that can determine SUV_max _by defining a ROI/VOI both manually and automatically within one examination. To our knowledge, none of the PET/CT manufacturers offer a software suite that can measure two matching uptakes in serial examinations automatically.

There are several studies on tumour delineation and quantification in PET/CT [6-12]. New quantitative approaches to evaluate treatment response have been suggested in some papers [6,11]. But differences in imaging parameters across PET centres have a negative effect on semi-automatic methods' performance [13,14]. Due to the increasing amount of examinations and the quantification that can be done, standardization of PET image acquisition and effective computer-aided quantification is warranted.

A common clinical setting for PET/CT is the evaluation of follow-up examinations after treatment or expectancy. Manual measurement of SUV_max _in single studies or changes in serial studies can be time consuming, making it difficult for clinicians and PET reviewers to follow-up all SUV_max _changes in serial studies in a comprehensive and effective way. Studies with semi-automatic quantification of SUV_max _changes in serial examinations suggest registration of images and SUV measurements are accurate and reliable [6,11]. Time saving qualities, or investigation of the reproducibility of using a semi-automatic approach, compared to manual reading of SUV_max _has not been elicited. The purpose of this study is to demonstrate these qualities using a semi-automatic method for calculation of SUV_max _of an abnormal lesion in serial studies when a region of interest (ROI) has been semi-automatically defined in the PET image.

## Materials and methods

### Patients

Patients with lung cancer or lymphoma who had undergone two PET/CT studies between July 2008 and January 2010 at the Skåne University Hospital in Malmö, Sweden were included retrospectively.

There were ten lung cancer patients and six lymphoma patients. Diagnoses were verified with biopsy in all cases except for one patient on which the diagnosis was based on clinical findings. The patients had not been treated with chemotherapy or radiation therapy for at least 6 months prior to the baseline examination. The time from baseline examination to the follow-up examination varied between 31 and 150 days. Follow-up examinations were performed to evaluate tumour progression in five cases and therapy response in eleven cases.

Pathological lesions with sharp contrast to surrounding areas, no formation of a large conglomerate mass and presence in both studies were selected. 16 patients (69% men, age 56 ± 15 (mean ± SD)) with 26 pathological lesions were included. Each patient had one to four pathological lesions for manual and semi-automatic measurement. The study was approved by the Research Ethics Committee at Lund University.

### Scan data

After 4-6 hours fast 4 MBq/kg of ^18^FDG was given intravenously. The effective dose was 5-7 mSv for PET, 2-3 mSv for low-dose CT and 10-15 for diagnostic CT. Data were acquired 60 min after injection and the patients were scanned from head to the upper thigh with the use of an integrated PET/CT system (Philips Gemini TF). A diagnostic/low dose CT scan obtained with the use of a standard protocol, 150 mAs/slice, 120 kV, a tube rotation time of 0.75 s per CT rotation, a pitch of 0.9, and a slice thickness of 5 mm preceded the PET scan covering the identical transverse field of view (a 2 min emission scan per table position and 6-10 bed positions per patient).

The PET image data sets were reconstructed iteratively with segmented correction for attenuation with use of the CT data. CT images, PET images and co-registered images were displayed by means of Philips workstation and program. Reconstructed PET and CT images were also transferred to a customized software developed for semi-automatic measurements.

### SUV_max _measurements

Three readers did manual and semi-automatic analysis of SUV_max _on 26 pathological lesions. One of the readers marked the 26 lesions in screenshots of coronal and transaxial PET images and these images were used during the study to secure that all three readers were measuring the same lesions with the manual and the semi-automatic method. The time to measure SUV_max _manually and semi-automatically, after complete loading of the serial studies, was recorded on all pathological lesions individually.

### Manual method

Manual measurements were done on a Philips PET/CT workstation (Philips Extended Brilliance Workspace, PET/CT Application Suite v1.5 K). The readers identified the lesions in both PET studies and used their most preferred software tool to determine the SUV_max_. Readers reported that they used the free-floating SUV search tool and ROIs.

### Semi-automatic method

A method to measure SUV_max _of a pathological lesion present in two studies from the same patient was developed. Transaxial PET and CT images from the two studies were used as input to the program. The two CT studies were automatically aligned by projecting the skeletons in the CT images on a 2D space. A rigid registration algorithm was used. CT images were reduced in size, blurred, thresholded and then overlaid to compare pixels with the formula: Σ_(i, j)_erf(i, j) = |IM_1_(i, j)-IM_2_(i, j)|

One of the images is repositioned and the formula is applied again to calculate the pixel error. The process is reiterated with less size reduction and blurring each time.

The reader marked an arbitrary pixel of a pathological lesion in one of the two PET studies. After this manual step the program automatically detects the corresponding lesion in the second PET study. The reader's mark in the first PET study has a corresponding location in the first CT study, provided that PET and CT images were correctly aligned from the PET/CT camera. The corresponding location in the second CT study was defined using the matching by the software and the corresponding location in the second PET study was defined.

**Figure 1:**
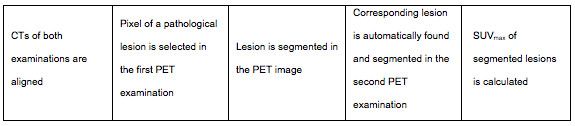
**Flowchart of the semi-automatic process**.

The volumetric segmentation of the pathological lesions was made in the PET images. The reader's mark in the first PET study and the corresponding location in the second PET study were used as seed points for the segmentation using the graph-cut algorithm [15]. SUV_max _in the entire lesion was then calculated automatically and the segmentations were presented to the reader. Semi-automatic analysis was done on a standard laptop computer (Figure 1).

### Statistical analyses

Intraobserver reproducibility was analysed with Intraclass correlation (ICC). A Bland-Altman analysis was used to assess the level of agreement between the two methods.

## Results

The Bland-Altman analysis indicates that the 95% limits of agreement between manual and semi-automatic assessment ranged from -2.67 to 1.29 (reader 1); -3.39 to 2.29 (reader 2); -2.21 to 1.18 (reader 3). The two methods consistently provide similar measures for most of the tumours. Limits of agreement ranged from ± 0.99 to ± 1.41 across the readers. Manual and semi-automatic method agreed in all cases whether SUV_max _had increased or decreased between the serial studies. The range of SUV_max _values for the manual method was 2.4 - 19.8 and for the semi-automatic method 2.6 - 21.4.

ICCs of SUV_max _readings between readers using the semi-automatic method in the first and second study were 1.00 (95% CI, 1.00-1.00) and 0.94 (95% CI, 0.89-0.97) respectively. ICC of SUV_max _readings between readers using the manual method were 1.00 (95% CI, 1.00-1.00) and 0.95 (95% CI, 0.90-0.97) for the first and second study respectively. Almost-perfect reproducibility was thus obtained with both methods.

**Figure 2:**
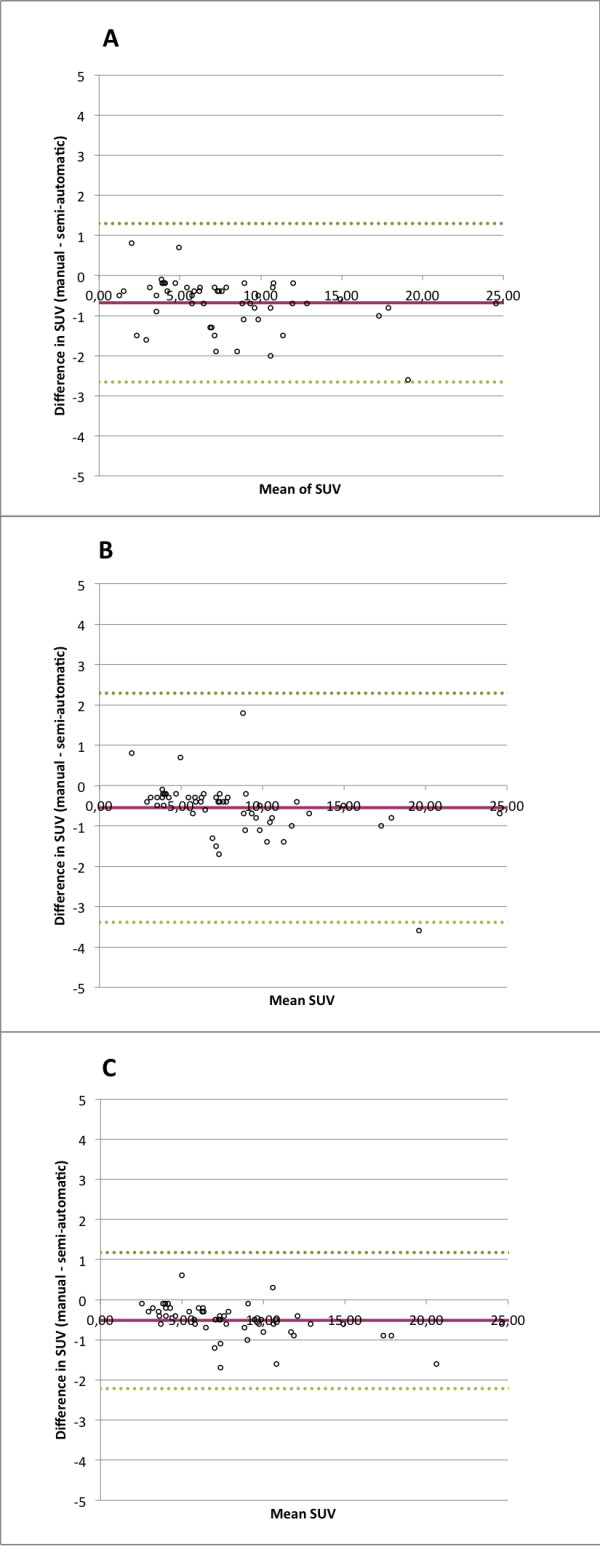
**Bland-Altman analysis of SUV_max _differences between both methods**. Limits of agreement for A: -2.67 to 1.29 (reader 1); B: -3.39 to 2.29 (reader 2); C: -2.21 to 1.18 (reader 3)

The average time to measure SUV_max _changes in two serial PET/CT examinations was significantly longer for the manual method compared to the semi-automatic method for all readers (Table 1). The semi-automatic method is up to 4 - 5 times faster compared to the manual method (Figure 2).

**Table 1:** Average time to measure changes in SUV_max _between two serial examinations for manual and semi-automatic methods and the difference between them

	Manual	Semi-automatic	Difference (95% CI)	*p*-value	X times faster
**Reader 1**	53.7 s	10.5 s	43.2 (36.4-49.9)	< 0.001	5
**Reader 2**	27.3 s	6.9 s	20.4 (13.6-27.1)	< 0.001	4
**Reader 3**	47.5 s	9.5 s	38.1 (31.3-44.8)	< 0.001	5

## Discussion

Good agreement between the two methods was showed with the Bland-Altman analysis. The somewhat wider range of limits of agreement in reader 2 (± 1.41) seems to be due to not finding the highest SUV_max _with the manual method within some large tumours with high SUV_max_. This resulted in a greater difference comparing to the semi-automatic method. Both methods agreed whether the SUV_max _had increased or decreased in the follow-up examination. The results of this study indicate the feasibility of using semi-automatic method in serial examinations.

Like other studies [5,6,9,10], the selection of tumours was limited to small-medium sized tumours, which were round, had a clear delineation on the CT scan and were not part of large conglomerates, which produce a "bleeding" effect on the PET image. The almost-perfect reproducibility obtained with both methods is likely dependent on the properties of the pathological sites. The finding supports the findings in previous studies [5,6,9,10]. Clinical reality provides many obstacles for semi-automatic segmentation and it remains to be seen how pathological sites can be best segmented by algorithms no matter the form, size or location it has.

Readers were not instructed whether they should perform the semi-automatic segmentation in the first or second study. In the feedback after measuring with both methods, readers agreed it was easier to segment the correct tumour of both examinations by selecting the tumour on the examination, which had the smallest size. Should the readers have been instructed only to segment in the first or second scan regardless, the results may have shown poorer segmentation quality.

Segmentation in the PET image has more advantages than segmentation in CT. In the study it enabled segmentation of lymphoma in the liver and in locations where the lesion in CT was adjacent to soft tissue. Also, the segmentation tool used did not adequately segment the lesions in CT.

Manual delineation capabilities were intentionally left out of the program in order to test the accuracy of semi-automatic segmentation and in order to keep the time of analysis to a minimum.

SUV_max _was chosen over SUV_mean _due to clinical praxis at our hospital when assessing PET/CT examinations. Furthermore, research has shown SUV_max _measurements are readily reproducible between readers [5]. The program shows systematically higher SUV_max _compared with the manual method. This may be due to different filter settings between the PET/CT manufacturer and the program. It is unlikely that all readers have systematically missed the "true" SUV_max _with the program.

## Conclusions

Good agreement was shown in absolute SUV_max _measurements between both methods. Almost-perfect reproducibility was seen between three readers using both semi-automatic and manual methods. Using semi-automatic method reduces time to calculate SUV_max _by up to 5 times. The findings show feasibility of using semi-automatic calculation of SUV_max _in serial studies and encourage further development of programs that accurately segments more complex pathological sites.

## Competing interests

The authors declare that they have no competing interests.

## Authors' contributions

JL contributed to patient recruitment, did manual reading, took part in data analysis and wrote the first version of the manuscript. SG contributed to patient recruitment and did manual reading. PH contributed to the development of the semi-automatic method and to data analysis. EJ contributed to the development of the semi-automatic method and performed the programming. SV contributed to development of the semi-automatic method, patient recruitment and did manual reading. LE conceived of the study and contributed to the design of the study, to development of the semi-automatic method and to data analysis. PW contributed to the design of the study and to data analysis. All authors read and approved the final manuscript.

## Funding

This research is supported by grants from ALF (all authors).

## Pre-publication history

The pre-publication history for this paper can be accessed here:

http://www.biomedcentral.com/1471-2342/12/6/prepub
